# Loss of Rsph9 causes neonatal hydrocephalus with abnormal development of motile cilia in mice

**DOI:** 10.1038/s41598-020-69447-4

**Published:** 2020-07-24

**Authors:** Wenzheng Zou, Yuqing Lv, Zux iang Liu, Pengyan Xia, Hong Li, Jianwei Jiao

**Affiliations:** 1 State Key Laboratory of Stem Cell and Reproductive Biology, Institute of Zoology, Chinese Academy of Sciences, Beijing, 100101 China; 2 University of Chinese Academy of Sciences, Beijing, 100049 China; 3 State Key Laboratory of Brain and Cognitive Science, Institute of Biophysics, The Innovation Center of Excellence on Brain Science, Chinese Academy of Sciences, Beijing, 100101 China; 4 State Key Laboratory of Membrane Biology, Institute of Zoology, Beijing, 100101 China; 5 Co-Innovation Center of Neuroregeneration, Nantong University, Nantong, 226001 China; 6 Innovertion Academy for Stem Cell and Regeneration, Chinese Academy of Sciences, Beijing, 100101 China; 7 Group of Neural Stem Cell and Neurogenesis, Institute of Zoology, Chinese Academy of Sciences, Beichen West Road, Chaoyang District, Beijing, 100101 China

**Keywords:** Developmental disorders, Developmental disorders, Ciliogenesis

## Abstract

Hydrocephalus is a brain disorder triggered by cerebrospinal fluid accumulation in brain cavities. Even though cerebrospinal fluid flow is known to be driven by the orchestrated beating of the bundled motile cilia of ependymal cells, little is known about the mechanism of ciliary motility. RSPH9 is increasingly becoming recognized as a vital component of radial spokes in ciliary “9 + 2” ultrastructure organization. Here, we show that deletion of the *Rsph9* gene leads to the development of hydrocephalus in the early postnatal period. However, the neurodevelopment and astrocyte development are normal in embryonic *Rsph9*^−/−^ mice. The tubular structure of the central aqueduct was comparable in *Rsph9*^−/−^ mice. Using high-speed video microscopy, we visualized lower beating amplitude and irregular rotation beating pattern of cilia bundles in *Rsph9*^−/−^ mice compared with that of wild-type mice. And the centriolar patch size was significantly increased in *Rsph9*^−/−^ cells. TEM results showed that deletion of *Rsph9* causes little impact in ciliary axonemal organization but the *Rsph9*^−/−^ cilia frequently had abnormal ectopic ciliary membrane inclusions. In addition, hydrocephalus in *Rsph9*^−/−^ mice results in the development of astrogliosis, microgliosis and cerebrovascular abnormalities. Eventually, the ependymal cells sloughed off of the lateral wall. Our results collectively suggested that RSPH9 is essential for ciliary structure and motility of mouse ependymal cilia, and its deletion causes the pathogenesis of hydrocephalus.

## Introduction

Hydrocephalus is a prevalent birth defect triggered by excessive accumulation of cerebrospinal fluid (CSF) in brain cavities^1^. Natural CSF flow is secreted by specialized ependymal cells in the choroid plexuses of lateral ventricles. In addition, the CSF carries signaling molecules, nutrients, microRNAs and exosomes^2–4^. It passes through the 3rd ventricles, cerebral aqueduct, and the 4th ventricles and can be absorbed by arachnoid granulations^5,6^. The directional flow of CSF is driven by continuous CSF secretion and by the orchestrated beating of bundles of motile cilia that are located at the apical surface of ependymal cells^7^.

The motile cilia are arranged structurally as a “9 + 2” axoneme, which is composed of nine interconnecting peripheral pairs of microtubules and a central pair of single microtubules. The radial spoke head protein, RSPH9, is a structural protein located at the T-shaped macromolecular configurations protruding from the nine peripheral pairs of microtubules^8^. Interaction between radial spoke heads and the central pair of single microtubules is the central for ciliary regulation. Previous studies showed that *Chlamydomonas* RSP9 mutant strains lack the entire radial spoke head complex and displacement of the central pair of single microtubules^9^. And zebrafish *Rsph9* mutant larvae exhibit similar cilia-dysmotility defects and reduced initiation of the acoustic startle response^10^. However, mutations in *RSPH9* cause primary ciliary dyskinesia (PCD; MIM 244400) in human, which is characterized by phenotypic heterogeneity and lacks a suitable “gold standard” diagnostic test^8,11,12^. In addition, it is difficult to build a model of direct protein interaction between radial spoke proteins and the central pair of single microtubules because of the gap between them. Thus, we further investigated the spatiotemporal developmental function of RSPH9 using mouse model.

In this study, we generated global knockout mouse models to elucidate the pathogenesis of PCD by targeting the murine *Rsph9* locus. We systematically investigated the development of *Rsph9*^−/−^ mice to understand the consequence of losing this gene. Our study reveals the role of RSPH9 in hydrocephalus pathogenesis and ependymal cilia motility in the developing mouse brain.

## Results

### Targeting *Rsph9* in mice

**Figure 1:**
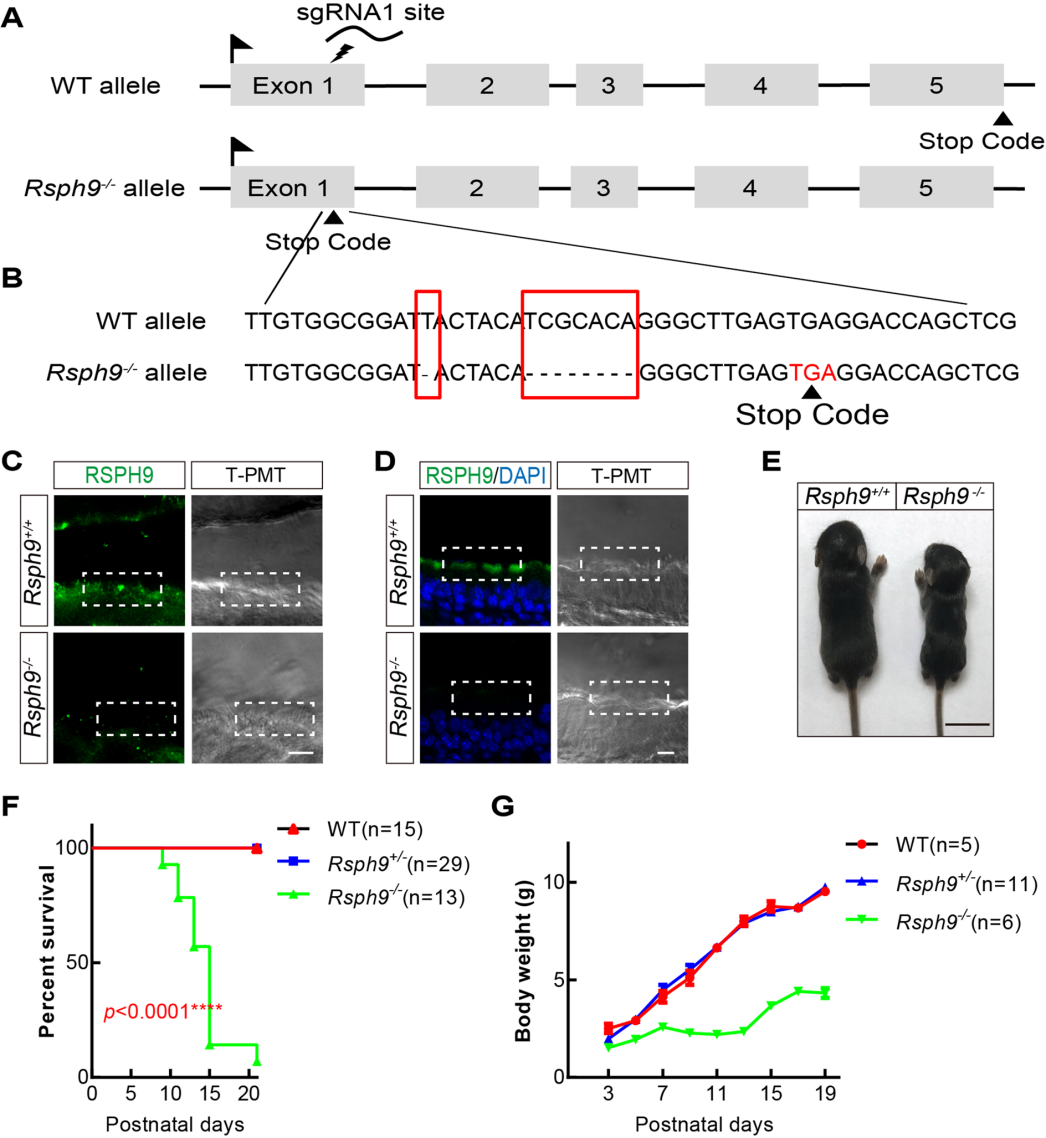
Generation of *Rsph9* knockout mice. (**A**) Schematic representation of the Rsph9 targeting strategy. The numbered boxes represent exons. sgRNA1 targets exon 1, leading to a stop codon that truncates the RSPH9 protein after the first exon. (**B**) Schematic drawing of the shearing of nucleotide bases. The red boxes represent deleted bases that resulted in frameshift mutations. (**C**) Immunofluorescence staining with RSPH9 in brain subventricular en-face of P7 mice. Representative cilia-containing regions are framed. T-PMT shows brightfield images taken by transmitted light detector. Scale bar, 5 μm. (**D**) Immunofluorescence staining with RSPH9 in trachea cilia of P7 mice. Representative cilia-containing regions are framed. Scale bar, 10 μm. (**E**) The survival rate of postnatal *Rsph9* mice recapitulated the phenotypes of wild-type, *Rsph9*^+/−^ and *Rsph9*^−/−^ mice. P values are generated by comparing between homozygous mutants and wild-type. (**F**) Body weight of wild type, *Rsph9*^+/−^ and *Rsph9*^−/−^ mice (data are expressed as the means ± SEMs). SEM is the standard deviation divided by the square root of the sample size. Student’s t test.

RSPH9-associated primary ciliary dyskinesia has a wide phenotypic variability in humans. To target *Rsph9* in mice, we used the CRISPR-Cas9 system and the zygote microinjection of a single-guide RNA1 targeting exon1 of *Rsph9* (Fig. 1A). The strategy deleted 8 base pairs to produce producing a premature stop codon at the end of exon 1, which significantly truncated the RSPH9 protein (Fig. 1B). The truncated RSPH9 with 61 amino acid residues are much shorter compared with normal RSPH9 with 276 amino acid residues. The generated heterozygous *Rsph9*^+/−^ mice were viable and fertile. We backcrossed them with C57BL/6 mice for more than five generations to obtain a more purified genetic background.

### Homozygous *Rsph9* mutations cause a slower growth rate and postnatal lethality

*Rsph9*^+/−^ mice were interbred to obtain homozygous knockout mice and were confirmed by genotyping PCR (Fig. S1A). The genotyping results on the first postnatal day revealed a prospective Mendelian ratio (1:2:1) and no sex-specific differences in survival. RSPH9 is highly expressed in multiciliated cells. Immunostaining with RSPH9 showed deletion of RSPH9 in *Rsph9*^−/−^ brain ependymal cilia and tracheal cilia (Fig. 1C,D). However, the knockout pups failed to grow normally, and most died during the weaning phase (Fig. 1E–G). Only a very few survived into adult (Fig. S1B). These *Rsph9*^−/−^ mice are characterized by enlarged dome-shaped skulls and severe neurological symptoms, including lethargy, apathy and muscle weakness.

### ***Rsph9***^−/−^ mice develop progressive hydrocephalus and sinusitis

**Figure 2:**
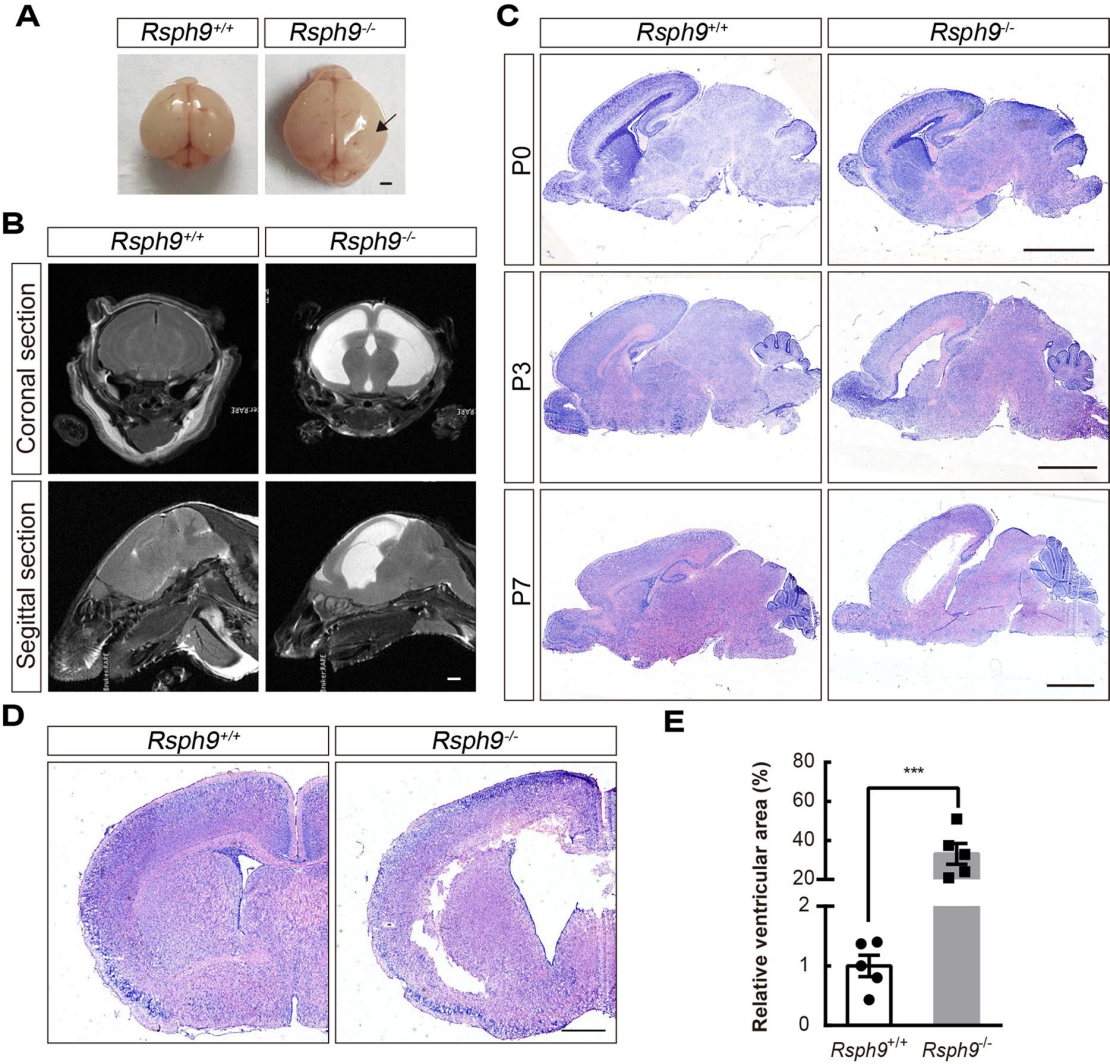
Severe postnatal hydrocephalus in *Rsph9*^−/−^ mice. (**A**) Images of *Rsph9*^+/+^ and *Rsph9*^−/−^ mouse brains at postnatal day 8 (P8). The arrow indicates the enlarged brain hemispheres. Scale bar, 100 μm. (**B**) T2-weighted coronal and sagittal magnetic resonance images (MRI) of the brains of *Rsph9*^+/+^ and *Rsph9*^−/−^ mice at P10. CSF in enlarged lateral ventricles is hyperintense. Scale bar, 100 μm. (**C**) Sagittal sections of P0 (top), P3 (middle) and P7 (bottom) mouse brains that were stained with Nissl. Scale bar, 200 μm. (**D**) Coronal sections of P8 mouse brains that were stained with Nissl. (**E**) Cerebral ventricular size in P8 brains (n = 5 mice per group; ***P < 0.001; data are expressed as the means ± SEMs). SEM is the standard deviation divided by the square root of the sample size. Student’s t test.

PCD is usually accompanied by randomized body laterality and respiratory disease. Therefore, we investigated these phenotypes in mice. There was no *situs inversus* in *Rsph9*^−/−^ mice, which shows RSPH9 is not associated with the determination of the left–right axis of visceral organs (Fig. S2A; n = 6). However, *Rsph9*^−/−^ mice developed severe neurological disorders and sinusitis (Fig. S2B). To analyze macrocephaly in *Rsph9*^−/−^ mice, the brains were isolated and were found to be enlarged (Fig. 2A). Magnetic resonance imaging revealed severe thinning of the cerebral cortex and enlarged hemispheres with massive accumulation of CSF in the lateral ventricles (Fig. 2B). The hippocampus and the hypothalamus were severely compressed. All of these are characteristic symptoms of hydrocephalus. To characterize the temporal feature of hydrocephalus in mice with the *Rsph9* deletion, we compared sagittal sections of the developing brain between P0 and P7 in wild-type and *Rsph9*^−/−^ mouse pups (Fig. 2C). There was no significant difference at P0. However, the area of lateral cerebral ventricular zone was comparatively increased at P3 and P7. Histological analysis of coronal brain sections revealed enlarged ventricles with abnormal brain morphology in P8 *Rsph9*^−/−^ mice (Fig. 2D,E). Cerebrospinal fluid analysis showed clear CSF and no visible brain hemorrhage, which indicated that hydrocephalus was not caused by hemorrhaging in the *Rsph9*^−/−^ mouse brain. Therefore, RSPH9 is not associated with *situs inversus*, but sinusitis. Furthermore, deletion of *Rsph9* can result in the development of brain dysfunction and progressive hydrocephalus during postnatal development in mice.

### Hydrocephalus is not caused by embryonic development defects in ***Rsph9***^−/−^ mice

**Figure 3:**
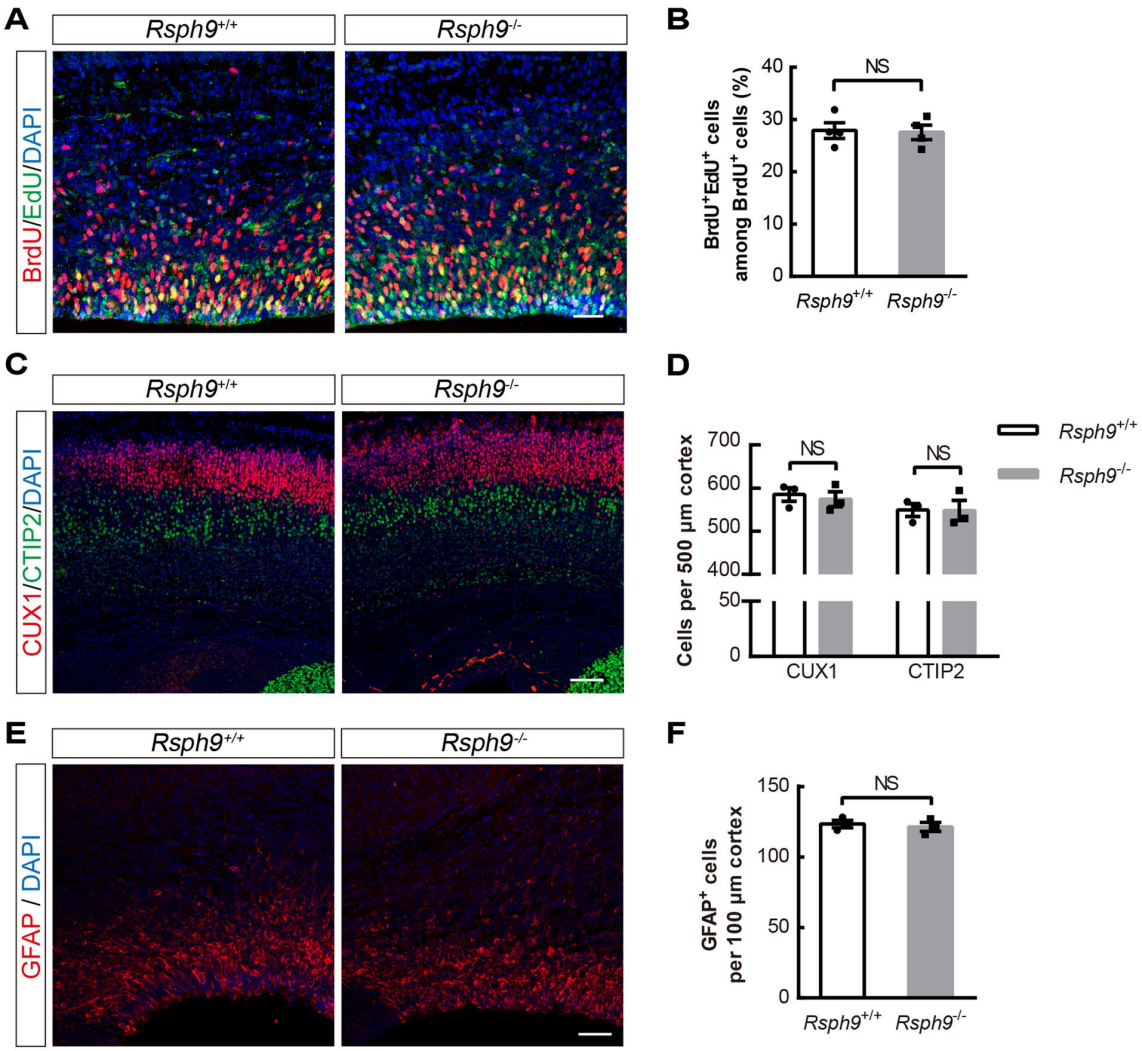
*Rsph9*^−/−^ mice develop normally in embryonic cortex development. (**A**) Developmental analysis for *Rsph9*^+/+^ and *Rsph9*^−/−^ mouse brains at embryonic day 15. BrdU (5-bromodeoxyuridin) and EdU (5-ethynyl-2′-deoxyuridine) were injected 24 h and 2 h, respectively, before sacrifice. Scale bar, 50 μm. (**B**) Quantification of the percentage of BrdU^+^ EdU^+^ cells among BrdU^+^ cells from *Rsph9*^+/+^ and *Rsph9*^−/−^ mice (n = 4 mice per group; NS, not significant; and data are expressed as the means ± SEMs). (**C**) Identification of cortical layers in wild type and *Rsph9*^−/−^ mice. CUX1 (red) is used to label layers II-IV, and CTIP2 (green) is used to label layer V. Scale bar, 100 μm. (**D**) Quantification of CUX1^+^ cells and CTIP2^+^ cells from *Rsph9*^+/+^ and *Rsph9*^−/−^ mice (n = 3 mice per group; *NS* not significant; and data are expressed as the means ± SEMs). (**E**) Immunofluorescence staining with a GFAP (astrocyte marker) antibody and DAPI (blue, cell nuclear marker) in P0 mouse brains. Scale bar, 50 μm. (**F**) Quantification of GFAP^+^ cells in the P0 *Rsph9*^+/+^ and *Rsph9*^−/−^ mouse cortex (n = 3 mice per group; NS, not significant; data are expressed as the means ± SEMs). SEM is the standard deviation divided by the square root of the sample size. Student’s t test.

Since ciliopathies are a group of genetic disorders closely related to neuronal cell fate, migration, and differentiation, as well as a host of adult behaviors^13^. We investigated whether hydrocephalus originates by embryonic brain developmental disorders. First, BrdU/EdU dual labeling experiments were conducted to confirm whether RSPH9 affects the neurogenesis process. The results showed that there was no significant difference in cell cycle dynamics in embryos at 15 days when comparing wild-type and *Rsph9*^−/−^ mice (Fig. 3A,B). Then the cortex markers CUX1 and CTIP2 were used for labeling layer II-IV and layer V of neonatal mice, which showed the typical cortical layer pattern of *Rsph9*^−/−^ mice (Fig. 3C,D). Neuronal migration is also not affected by *Rsph9* deletion. Immunofluorescent staining of glial fibrillary acidic protein (GFAP) was used to label astrocytes, and we found no significant change in GFAP-positive cell number or expression pattern between P0 *Rsph9*^+/+^ and *Rsph9*^−/−^ mice (Fig. 3E,F). Taken together, neurogenesis, neuronal migration and astrocyte development were determined to be undisturbed by *Rsph9* deletion in mice. Hydrocephalus was caused by postnatal developmental defects.

### CSF flow and circulation are impaired in *Rsph9*^−/−^ mice

**Figure 4:**
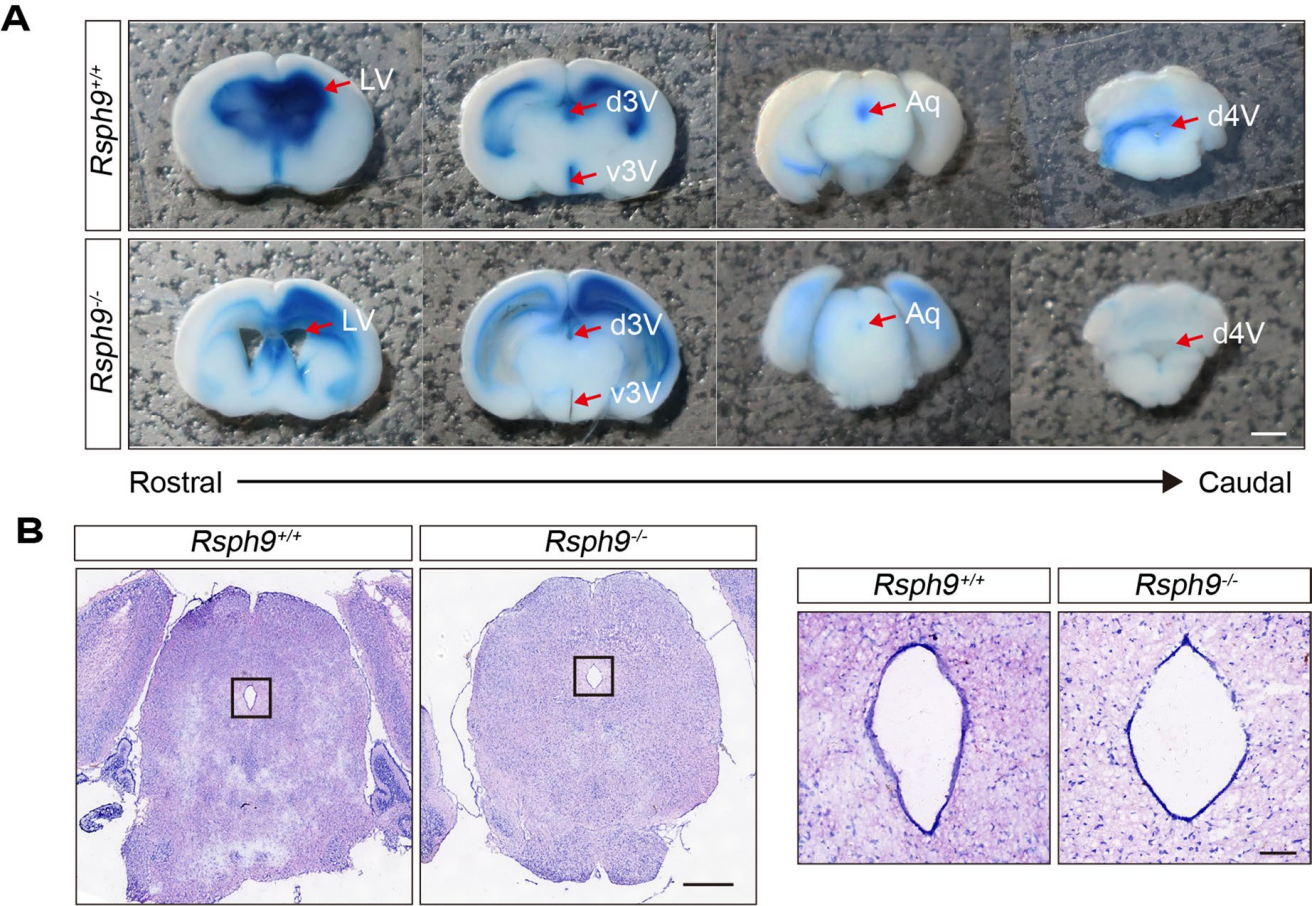
The reduction of CSF flow in *Rsph9*^−/−^ mice. (**A**) CSF flow analysis in P8 *Rsph9*^+/+^ and *Rsph9*^−/−^ mice. Evans blue dye was injected into the anterior horn of the lateral ventricle (LV). The dye was allowed to flow with CSF in vivo for 10 min. Coronal sections showed that the dye was detected in the third and fourth ventricles in *Rsph9*^+/+^ mice, but the same was not observed in *Rsph9*^−/−^ mice. *d* dorsal, *v* ventral, and *Aq* central aqueduct. Scale bar, 2 mm. (**B**) Nissl staining of *Rsph9*^+/+^ and *Rsph9*^−/−^ mouse brains at P8 showing that there is no aqueductal stenosis in *Rsph9*^−/−^ mice. Scale bars: 1 mm (left), 2 mm (right).

Cerebrospinal fluid is produced by the choroid plexus in lateral ventricles passing through the foramina of Monro, the 3rd ventricle, the cerebral aqueduct and the 4th ventricle. We injected Evans blue dye into the right lateral ventricle to investigate CSF flow through the ventricular system in *Rsph9*^−/−^ mice. In wild-type mice, the tracer could travel through the third ventricle, central aqueduct and fourth ventricle 10 min after injection (Fig. 4A; the upper row, n = 3). In contrast, no tracer or only very little tracer could be detected at the third ventricle and the fourth ventricle in *Rsph9*^−/−^ mice (Fig. 4A; the lower row, n = 4). Nissl staining results showed that the shape of the central aqueduct in P8 *Rsph9*^−/−^ mice was intact, the size was unchanged, and no blockage occurred (Fig. 4B). Thus, these results indicate that the barrier of CSF flow is due to the disrupted activity of the ependymal cells.

### ***Rsph9***^−/−^ ependymal cells display defects in motion pattern of cilia bundles

**Figure 5:**
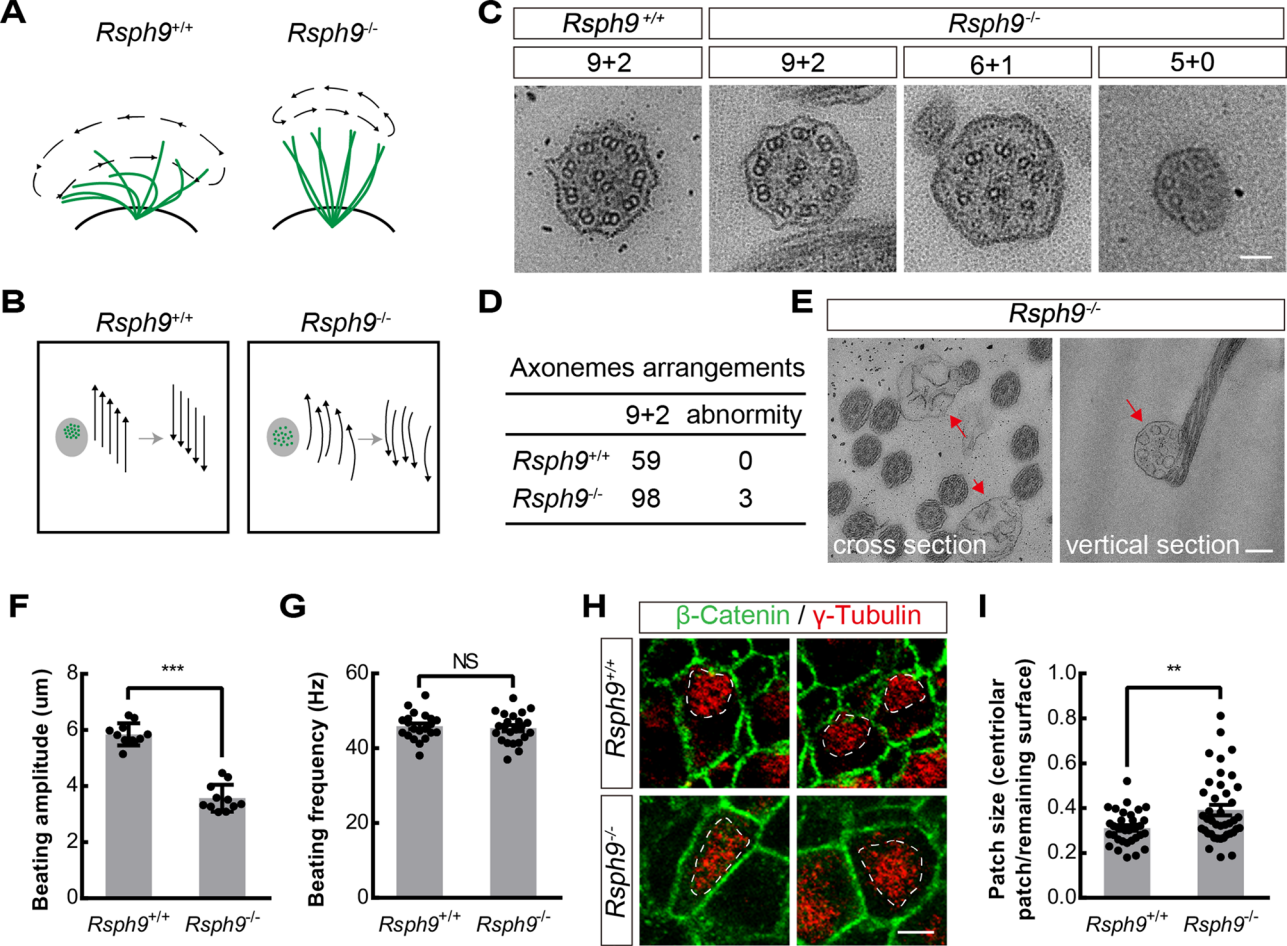
*Rsph9*^−/−^ ependymal cells display defects in motion pattern of cilia bundles. (**A**) Diagram of wild type ciliary beat cycle and Rsph9^−/−^ ciliary beat cycle from the side views. Wild type ciliary beat pattern is characterized by a strong beat stroke and a recovery stroke. Rsph9^−/−^ ciliary beat pattern is characterized by gently stroke from side to side. (**B**) Diagram of wild type ciliary beat cycle and Rsph9^−/−^ ciliary beat cycle from the top views. Wild type is characterized by orderly planar beating pattern. Rsph9^−/−^ is characterized by disorderly rotation beating pattern. (**C**) TEM images of ependymal cilia in wild-type and *Rsph9*^−/−^ mice. WT mice presented normal axonemes with “9 + 2” ultrastructure. Some *Rsph9*^−/−^ motile cilia exhibited a “9 + 2” ultrastructure, but others exhibited a disorganized axonemal structure. Scale bar, 50 nm. (**D**) Quantification results for TEM-cross sections of *Rsph9*^+/+^ and *Rsph9*^−/−^ ependymal cilia. (**E**) The ectopic abnormal ciliary membrane inclusions in the *Rsph9*^−/−^ (red arrows). Scale bar, 200 nm. (**F**) Quantification of cilia beating amplitude (n = 11 cilia per group; ***P < 0.001; and data are expressed as the means ± SEMs). (**G**) Quantification of cilia beating amplitude (n = 21 cilia for wild-type, n = 19 cilia for *Rsph9* mutants; NS; and data are expressed as the means ± SEMs). (**H**) Immunofluorescence staining with antibodies for β-Catenin antibody (greed, adherens junction) and γ-tubulin (red, centrioles) in wholemounts of lateral ventricular walls at P7. Centriolar patches are outlined with dashed white lines. Scale bars, 5 μm. (**I**) Quantification of ratio of centriolar patch size and total cell surface (n = 34 cells for wild-type, n = 41 cells for *Rsph9* mutants; **P < 0.01; and data are expressed as the means ± SEMs).

To assess the disorder of ependymal cells, whole-mount staining of the subventricular zone en-face was conducted to show the cilia bundles in the wild-type and *Rsph9*^−/−^ mouse brain (Fig. S3A,B). Wholemount immunostaining for β-catenin revealed intact adherens junctions in *Rsph9*^−/−^ mice (Fig. S3C). To evaluate the pattern of ependymal cilia beating in *Rsph9*^−/−^ ependymal cilia, video microscopy experiments were conducted. The analysis results revealed that the movement of beating cilia bundles is characterized by lower amplitude from the side views in P7 *Rsph9*^−/−^ mice compared with that of wild-type mice (Movie 1, 2, Fig. 5A,F). The wild-type cilia bundles moved orderly with planar beating pattern from the top views (Movie 3, Fig. 5B). The *Rsph9*^−/−^ cilia bundles, by contrast, moved disorderly with rotation beating pattern (Movie 4, Fig. 5B). Beating frequency was not significantly affected in *Rsph9*^−/−^ mice (Fig. 5G). Transmission electron microscopy (TEM) was performed on ependymal cilia to investigate the ciliary axoneme ultrastructure. In wild-type ependymal cilia, all axonemes exhibited a typical “9 + 2” ultrastructure (Fig. 5C). In *Rsph9*^−/−^ ependymal cilia, most axonemes exhibited normal ultrastructure and few exhibited various defects (Fig. 5C,D). The central pair of microtubules and the outer microtubule doublets may turn into single microtubules or may become vacant. Moreover, the *Rsph9*^−/−^ cilia frequently had abnormal ectopic ciliary membrane inclusions (Fig. 5E). Thus, RSPH9 has no significant effect on the “9 + 2” arrangement of microtubules. Furthermore, immunofluorescence analysis showed that assembling of RSPH3 into radial spoke head complex is not affected by RSPH9 deletion (Fig. S4). The defects in *Rsph9*^−/−^ cilia may due to disorders of sliding motion between adjacent microtubules. Remarkably, centriolar patch size was significantly increased in *Rsph9*^−/−^ cells (Fig. 5H,I). The mechanical stress of cilia beating pattern of *Rsph9*^−/−^ cells may destroy apical centriolar distribution. These results show that RSPH9 is necessary for coordinated beating of ependymal cilia. Defects in *Rsph9*^−/−^ ependymal cells can lead to disruption of the pattern of cilia beating and can give rise to hydrocephalus.

### Hydrocephalus in ***Rsph9***^−/−^ mice results in astrogliosis, microgliosis, cerebrovascular abnormality and myelination disorders

**Figure 6:**
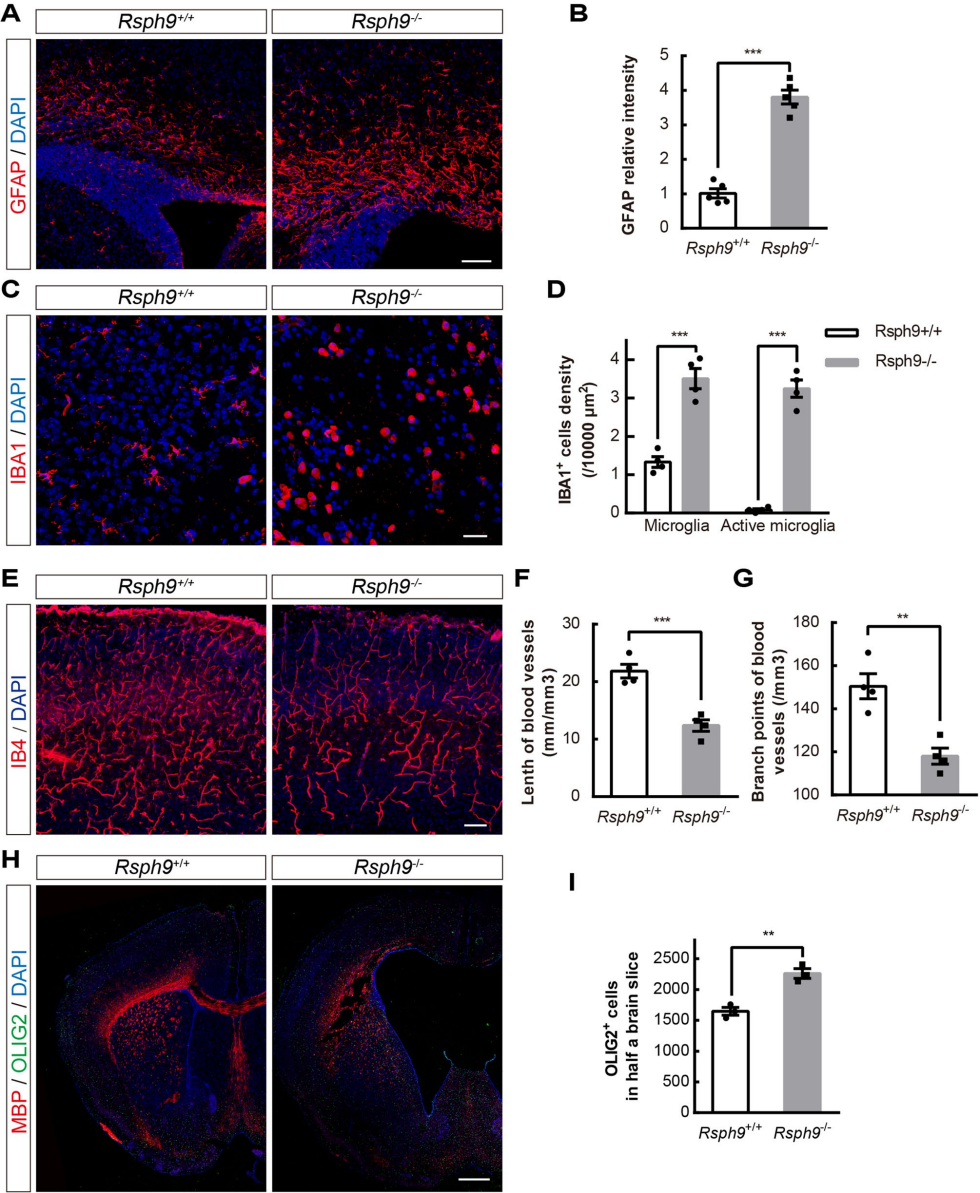
The hydrocephalus in *Rsph9*^−/−^ mice developed severe pathological reactions. (**A**) Immunofluorescence staining with antibodies for GFAP (red, astrocyte marker) and staining for DAPI (blue, nuclear marker) in P8 mouse brains. Scale bars, 100 μm. (**B**) Quantification of reactive gliosis in *Rsph9*^+/+^ and *Rsph9*^−/−^ mice (n = 5 mice per group; ***P < 0.001; and data are expressed as the means ± SEMs). (**C**) Immunofluorescence staining with antibodies for IBA1 (red, microglia marker) and staining for DAPI (blue, nuclear marker) in P8 mouse brains. Scale bars, 50 μm. (**D**) Quantification of total microglia and reactive microglia from *Rsph9*^+/+^ and *Rsph9*^−/−^ mice (n = 4 mice per group; ***P < 0.001; and data are expressed as the means ± SEMs). (**E**) Immunofluorescence staining with antibodies for IB4 (red, vessel marker) and staining for DAPI (blue, nuclear marker) in P8 mouse brains. Scale bars, 100 μm. (**F**,**G**) Quantification of vessel density and branching frequency from *Rsph9*^+/+^ and *Rsph9*^−/−^ mice (n = 4 mice per group; **P < 0. 01; ***P < 0.001; and data are expressed as the means ± SEMs). (**H**) Immunofluorescence staining for MBP (red, myelinating glia marker), OLIG2 (green, mature oligodendrocyte marker) and DAPI (blue, nuclear marker) in P8 mouse brains. Scale bar, 500 μm. (**I**) Quantification of OLIG2^+^ cells in P8 *Rsph9*^+/+^ and *Rsph9*^−/−^ mouse brains (n = 3 mice per group; **P < 0. 01; and data are expressed as the means ± SEMs). SEM is the standard deviation divided by the square root of the sample size. Student’s t test.

Hydrocephalus can damage brain tissue and cause a wide range of symptoms. Here we investigated the pathological characteristics of the thinning of cerebral cortex. The hydrocephalus caused by *Rsph9* deletion was accompanied by astrogliosis and microgliosis in the cortex. Immunostaining with GFAP in the P8 *Rsph9*^−/−^ cerebral cortex was much stronger than that in the control mice (Fig. 6A,B). Immunostaining with ionized calcium binding adaptor molecule 1 (IBA1) showed that the microglia increased dramatically with the severity of hydrocephalus (Fig. 6C,D). The microglia were almost all activated characterized by shorter processes and larger soma in P8 *Rsph9*^−/−^ mice. This shows that hydrocephalus is proceeded by the inflammatory response. The analysis of cerebral vessels showed that vessel density and branching were reduced in P8 *Rsph9*^−/−^ mice (Figs. 6E–G and S5). Hydrocephalus in *Rsph9*^−/−^ mice also attenuated the expression of myelin basic protein (MBP), which is a marker of myelinating glia, but it enhanced the expression of oligodendrocyte transcription factor 2 (OLIG2), which is a marker of oligodendrocyte progenitor cells and mature oligodendrocytes (Fig. 6H,I). It turned out that myelin is damaged in *Rsph9*^−/−^ mice and that enhanced OLIG2 expression may contribute to myelin repair. Immunostaining with an ependymal layer marker (S100β) showed slightly rupture of the ependymal layer in P8 *Rsph9*^−/−^ mice (Fig. S6A). And then the ependymal layers were severely damaged and ependymal exfoliation was detected in P12 *Rsph9*^−/−^ mice (Fig. S4B). Altogether, these results suggest that in P8 *Rsph9*^−/−^ mice, hydrocephalus is associated with severe pathological reactions, inflammation reactions and myelination disorders.

## Discussion

RSPH9 is known to be a component of the axonemal radial spoke head complex, which is a thin stalk attached to the outer doublet microtubule in motile cilia. In this study, we show that *Rsph9*-deficient mice developed severe hydrocephalus with postnatal ventriculomegaly and severe sinusitis. The characteristic feature of hydrocephalus is the excessive CSF in ventricular dilation. Clinically, stenosis and obliteration of the aqueduct often initiate the pathogenesis of hydrocephalus^14–17^. Several cilia related genes have been accounted for by hydrocephalus. Genetic deficiency of genes, such as *Ccdc39*^18^, *Jhy*^19^, *Ulk4*^20^, *Lrrc6*^21^, *Zmynd10*^22^, and *Tap73*^23^, may lead to defects in ependymal differentiation or obstruction of aqueducts. However, our results showed that neurodevelopment and astrocyte development are normal in embryonic *Rsph9*^−/−^ mice. Although the Evans blue dye tracing experiment demonstrated defects in directional CSF flow, the tubular structure of the central aqueduct was comparable in *Rsph9*^−/−^ mice. Additionally, as hydrocephalus in murine models of ciliary gene knockouts is background dependent, we backcrossed the first generation with C57BL/6 mice for more than five generations to obtain a more purified genetic background.

Ciliated ependymal cells can generate and maintain complex, spatiotemporally regulated flow networks^7^. The beating pattern and beating frequency of ependymal cilia are spatially different^24,25^. Our video microscopy results showed that the movement of beating cilia bundles is characterized by lower amplitude and disorderly rotation beating pattern instead of orderly planar beating pattern in subventricular zone en-face of *Rsph9*^−/−^ mice. Liu et al. reported that knockdown of RSPH9 by RNAi in mouse ependymal cells, which differentiated from radial glia ex vivo, resulted in a near complete CP loss^26^; however, our results showed that there were no defects with CP in *Rsph9*^−/−^ ependymal motile cilia in vivo, and few exhibited various ultrastructure disorganizations. This may be due to the truncated RSPH9, which may still support the central pair formation, or the differences between in vivo and in vitro experiments. Furthermore, our results showed that centriolar patch size was significantly increased in *Rsph9*^−/−^ cells. Structural changes of radial spoke complex in RSPH9 deletion likely disturb doublet microtubules to tolerate both tensile and compressive stresses during ciliary beating^27^. This may disrupt the mechanical resistance of the apical actin network around centrioles, in turn, and disrupt centriole stability^28^.

Hydrocephalus proceeds with reactive astrogliosis and microgliosis, which lead to the formation of glial scars. Upon activation by injury, active glial cells release chemokines and cytokines, which help recruit of microglia^29,30^. Recruitment facilitates the formation of glial scars, which impede neovascularization and block the growth of neuronal processes^31^. Our experimental data are consistent with previous results^29,32–34^. We have provided further evidence that vessel density and branching frequency are both decreased in mice with hydrocephalus. Furthermore, it has previously been reported that oligodendrocyte precursor migration is associated with the abluminal endothelial surface of nearby blood vessels^35^, which may explain the defects of myelin and the accumulation of OLIG2^+^ cells in the medial ganglionic eminence of *Rsph9*^−/−^ mouse brains.

Dysfunction of RSPH9 can change the motion pattern of motile cilia. However, we still do not know how this change occur in *Rsph9*^−/−^ ciliary motility. Our knowledge of the mechanism of ciliary organization and motility has been very limited. In our experiments, we cannot see the ultrastructure of radial spoke complex clearly using TEM, and there remains questions of that whether RSPH9 affects localization of other radial spoke head proteins. The precise ultrastructural organization and sliding mechanism of radial spoke complex are still need to be investigated.

## Methods

### Mice

All mice were housed in specific pathogen-free conditions and maintained on a 12:12 h light–dark lighting cycle, with lights off at 19:00. Animal procedures were performed in accordance with experimental protocols and approved by Animal Care and Use Committees of the Institute of Zoology, Chinese Academy of Sciences. The Experimental Animal Center of the Institute of Zoology generated the Rsph9 knockout mice. The *Rsph9* knockout mice were generated by C57Bl/6 × 129/SvEv zygote microinjection with CRISPR-Cas9 system. Heterozygous *Rsph9*^+/−^ mice were back-crossed to C57BL/6 mice for at least five generations.

### Antibodies

For immunofluorescence analysis, the following primary antibodies were used: mouse anti-ARL13B (1:1,000 dilution, Abcam, #Ab136648), Goat anti-IBA1 (1:500, Abcam, #Ab5076), mouse anti-IB4 (1:400, Vector Laboratories, #B-1205), rabbit anti-GFAP (1:3,000, Dako, #Z0334), rabbit anti-S100β (1:1,000, Proteintech, #15146-1-AP), rat anti-BrdU (1:1,000, Abcam, #ab6326), rabbit anti-CUX1 (1:500, Santa Cruz Biotechnology, #sc-13024), rat anti-CTIP2 (1:500, Abcam, #ab18465), rabbit anti-RSPH9 (kindly gifted from Zhu Xueliang, Shanghai Institute of Biochemistry and Cell Biology, CAS), rabbit anti-RSPH3 (1:1,000, Proteintech, #17603-1-AP).

### Histology and immunofluorescence confocal microscopy

Tissues were fixed in 4% paraformaldehyde (PFA) overnight and dehydrated in 30% sucrose, and 15 μm-thick cryosections were prepared. For Nissl staining, 0.1% Cresyl Violet solution was used. For immunofluorescence, sections were blocked by 5% boveine serum albumin/0.1% Triton X-100/PBS for 1 h, incubated with primary antibodies at 4 °C overnight and fluorophore-conjugated secondary antibodies for 2 h. Images were taken with a Zeiss LSM780 laser scanning confocal microscope and Leica Aperio VESA8 microscope.

### Transmission electron microscopy (TEM)

The medial walls of P6 forebrains were fixed in 4% PFA. The samples were cut into 300 μm thick by Leica vibratome. The brain slices were shaped into cubes and fixed in electron microscopy grade 2% PFA and 2.5% glutaraldehyde in PBS (pH 7.4) at 4 °C overnight. Tissues were washed with PBS three times, and post-fixed in 1% osmium oxide for 1 h. The samples were thoroughly washed in PBS and dehydrated through an ethanol series (30%, 50%, 70%, 80%, 90%, 95%, 100%). The samples were washed twice in 100% acetone. Soak the samples in 1:1 ratio of acetone to epoxy resin for 1 h, then in 1:3 ratio of acetone to epoxy resin for 3 h, and in pure epoxy resin for more than 5 h. The samples were embedded and polymerized in epoxy resin at 60 °C for 48 h. Ultra-thin sections of 60 nm were obtained with Leica UC7 ultramicrotome, stained with uranyl acetate and Reynold’s lead citrate, and imaged using a transmission electron microscope (Tecnai G2 F20 TWIN TMP).

### Video microscopy of ciliary motion

P7 wild-type and Rsph9^−/−^ brains were collected and dissected in DMEM/F12 supplemented with l-glutamine and 2% B27 (Invitrogen) at room temperature. 200 μm-thick sections including ventromedial walls of lateral ventricle was acquired using a Leica vibratome. Images were acquired by a customized microscopy with a Nikon S Plan Fluro 40 × objective. The motion of cilia was captured for 7 s (380 frames/s) with a high-speed CMOS camera (PDV, MV-500C). Time-series images were captured at a resolution of 640 by 480 pixels and saved in raw format with timing information. A MATLAB (Mathworks, ver 2015b) script was used to select the region of interest (ROI) and export the ROI into PNG format. The in plane drifts due to the environmental vibrations were corrected by co-registering the PNG image series to the first image with TurboReg (ImageJ).

### Cerebral ventricular injection of tracers (systemic CSF flow analysis)

The pattern of CSF flow is practically the same as the one described by^20^. Briefly, four *Rsph9*^−/−^ and five controls postnatal day 8 mice were anesthetized. Then slowly injecting 5 μl Evans blue dye (4% in PBS) into the lateral ventricle at 1.7 mm posterior, 0.8 mm right and 1.8 mm deep from the Bregma on the head. The mice were sacrificed 10 min later. Whole brains with a part of the spinal cord were fixed in 4% PFA overnight. 1 mm-thick coronal slices were generated by Vibrating Microtomes Tissue Slicer.

## Supplementary information

Supplementary Information.

Supplementary Video 1.

Supplementary Video 2.

Supplementary Video 3.

Supplementary Video 4.
