# Supplementary material for: Loss of Rsph9 causes neonatal hydrocephalus with abnormal development of motile cilia in mice
Source: Sci Rep. 2020 Jul 24;10:12435. doi: 10.1038/s41598-020-69447-4 (PMC7382491; doi:10.1038/s41598-020-69447-4)
Supplement: Supplementary file 1 — Supplementary Information. [file 41598_2020_69447_MOESM1_ESM.pdf]

Loss of Rsph9 causes neonatal hydrocephalus with abnormal development of motile cilia in mice

Wenzheng Zou, Yuqing Lv, Zuxiang Liu, Pengyan Xia, Hong Li, Jianwei Jiao

Supplementary figures

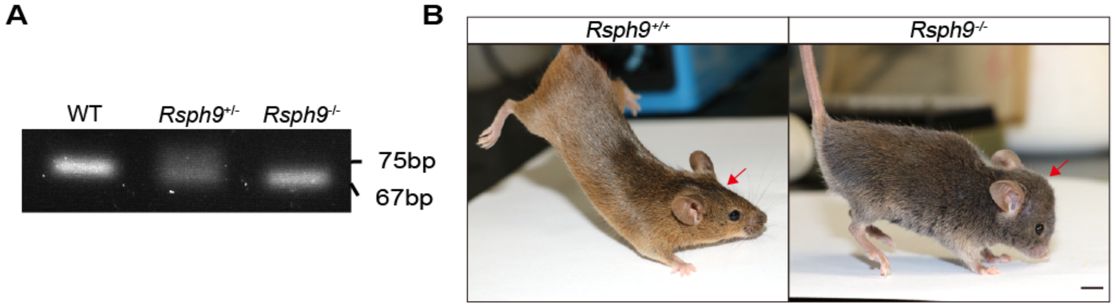

Figure S1. The *Rsph9*$^{-/-}$ mice developed macrocephaly

(A) Genotyping of wild-type, *Rsph9*$^{+/-}$ and *Rsph9*$^{-/-}$ mice. PCR products were 75 bp and 67 bp from *Rsph9*$^{+/+}$ and *Rsph9*$^{-/-}$ mice, respectively. There were both 75 bp and 67 bp PCR products from *Rsph9*$^{+/-}$ mice.

(B) Images of *Rsph9*$^{+/+}$ and *Rsph9*$^{-/-}$ mice at 3 months. Arrows indicate the dome-shaped skull. Scale bar, 1 cm.

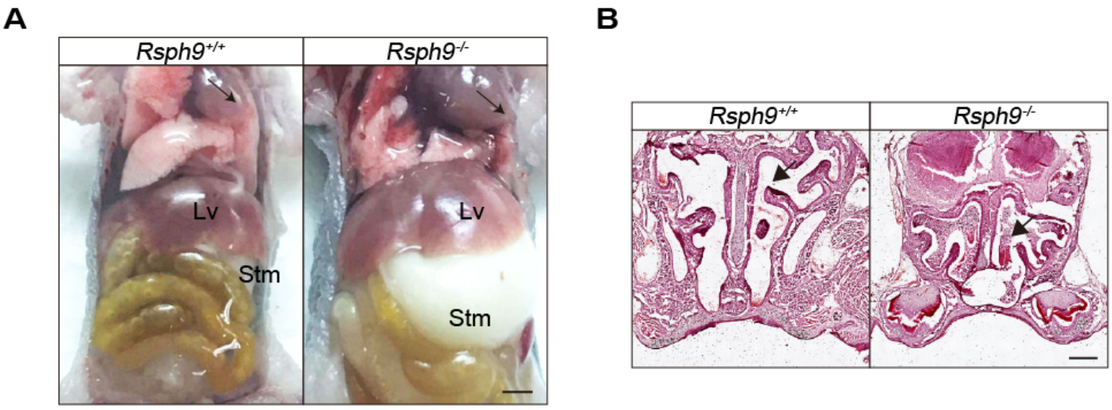

Figure S2. The *Rsph9*$^{-/-}$ mice developed situs solitus and sinuses

(A) No *situs inversus* was observed in *Rsph9*$^{-/-}$ mice. The right-sided stomach, heart and liver were found in 6 knockout mice at P4. Scale bar, 100 μm.

(B) Coronal section of the nasal region of wild-type and *Rsph9*$^{-/-}$ mice. The arrow shows the sinuses of *Rsph9*$^{-/-}$ mice were filled with pus. Scale bar, 500 μm.

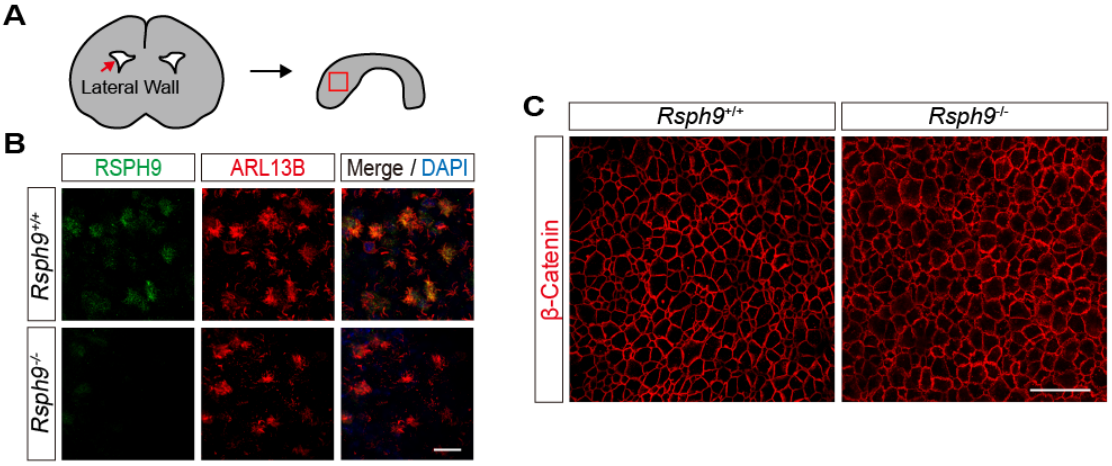

Figure S3. Wholemounts staining on the lateral wall

(A) Schematic representation of the lateral ventricular wall (indicated by red arrow) and the region of interest (red square).

(B) Immunofluorescence staining with RSPH9 (green) and ARL13B (red) antibodies in the ependymal cells of P6 mice. Scale bar, 20 µm.

(C) Immunofluorescence staining with β-Catenin antibody in P8 mouse brains. Wholemounts of the lateral wall of brain ventricles indicate the adherens junctions between ependymal cells. Scale bar, 50 µm.

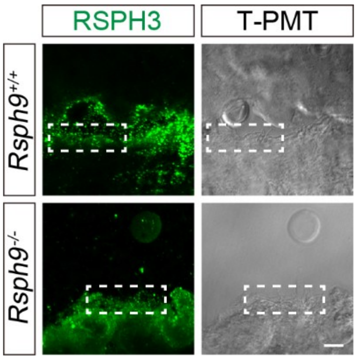

Figure S4. Deletion of Rsph9 does not affect assembly of RSPH3

Immunofluorescence staining for RSPH3 in P7 *Rsph9*$^{+/+}$ and *Rsph9*$^{-/-}$ ependymal cells. Representative cilia-containing regions are framed. Scale bar, 5 μm.

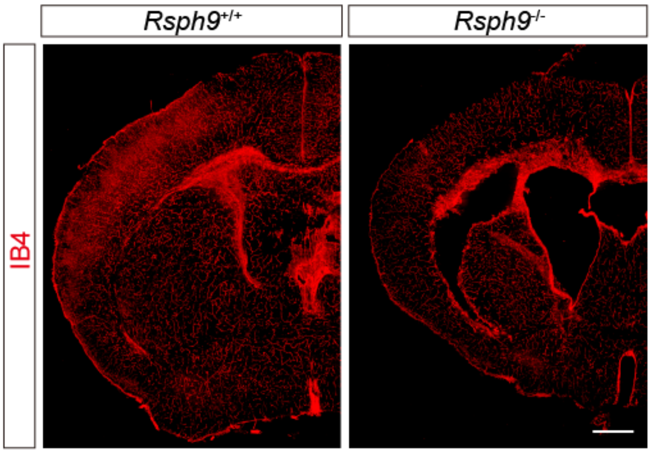

Figure S5. Cerebrovascular dysplasia in *Rsph9*$^{-/-}$ mice

Immunofluorescence staining for IB4 (vessel marker) in P8 mouse brains. Scale bar, 500 μm.

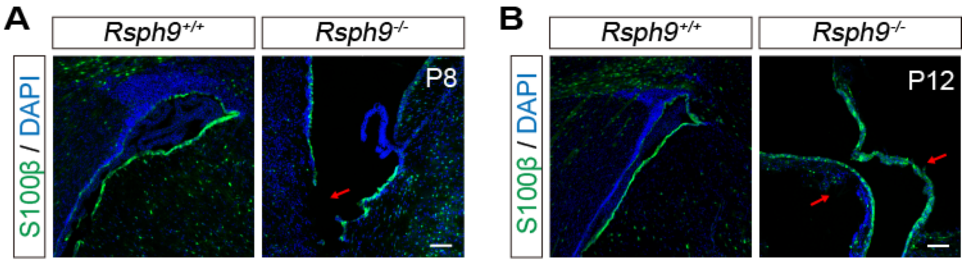

Figure S6. The ependymal layers were disrupted in *Rsph9*$^{-/-}$ mice

(A) Immunofluorescence staining with an S100β (green, ependyma marker) antibody and DAPI in P8 *Rsph9*$^{+/+}$ and *Rsph9*$^{-/-}$ mouse brains. The arrow indicates rupture of the ependymal layer. Scale bar, 100 μm.

(B) Immunofluorescence staining for S100β and DAPI at P12. The arrows indicate the shedding ependymal layer from subventricular zone. Scale bar, 50 μm.

Movie 1. Recording of ependymal cilia from the side view in the P7 wild-type mouse.

The cilia bundles display a strong beating stroke followed by a recovery stroke (video captured at speed of 380 frames per second and played back at 18 frames per second).

Movie 2. Recording of ependymal cilia from the side view in the P7 *Rsph9*$^{-/-}$ mouse.

The cilia bundles display gently stroke from side to side (video captured at speed of 380 frames per second and played back at 18 frames per second).

Movie 3. Recording of ependymal cilia from the top view in the P7 wild-type mouse.

The cilia bundles display orderly planar beating pattern (video captured at speed of 380 frames per second and played back at 18 frames per second).

Movie 4. Recording of ependymal cilia from the top view in the P7 *Rsph9*$^{-/-}$ mouse.

The cilia bundles display disorderly rotation beating pattern (video captured at speed of 380 frames per second and played back at 18 frames per second).
